# Lloyd’s Maps

**Published:** 1874-12

**Authors:** 


LLOYD'S MAPS.—Lloyd, the celebrated maker of maps, who furnished Gen Grant and the Union Army with maps, has invented a process of printing from steel Lloyd's Map of American Continent—showing from ocean to ocean—on one entire sheet of bank note paper, 40x50 inches large, on a lightning press, and colored, sized and varnished for the wall so as to stand washing, and mailing anywhere in the world for 25 cents, or unvarnished for 10 cents. This map shows the whole United States and Territories in a group, from surveys to 1875, with a million places on it, such as towns, cities, villages, mountains, lakes, rivers, streams, gold mines, &c. This map should be in every house. Send 25 cents to the Lloyd Map Company, Philadelphia, and you will get a copy by return mail.

